# Kidney Transplantation in Patients With Erdheim-Chester Disease

**DOI:** 10.1016/j.ekir.2025.103763

**Published:** 2026-01-02

**Authors:** Giulia Palazzini, Francesco Pegoraro, Jean-François Emile, Sanjeev Akkina, Elisa Buti, Anna Perrone, Corrado Campochiaro, Lorenzo Dagna, Edoardo La Porta, Decimo Silvio Chiarenza, Anna J. Peired, Achille Aouba, Raphaële Renard-Penna, Julien Haroche, Augusto Vaglio

**Affiliations:** 1 Department of Biomedical Experimental and Clinical Sciences "Mario Serio", University of Florence, Florence, Italy; 2 Nephrology and Dialysis Unit, Meyer Children’s Hospital IRCCS, Florence, Italy; 3 Department of Hematology and Oncology, Meyer Children’s Hospital IRCCS, Florence, Italy; 4 Department of Experimental and Clinical Medicine, University of Florence, Florence, Italy; 5 Paris-Saclay University, Versailles SQY University, Assistance Publique-Hôpitaux de Paris, Ambroise-Paré Hospital, Smart Imaging, Service de Pathologie, Boulogne, EA4340-BECCOH, France; 6 Loyola University Medical Center, Maywood, Illinois, USA; 7 Pediatric Radiology Department, Meyer Children’s Hospital IRCCS, Florence, Italy; 8 Vita-Salute San Raffaele University, Milan, Italy; 9 Unit of Immunology, Rheumatology, Allergy, and Rare diseases, IRCCS San Raffaele Scientific Institute, Milan, Italy; 10 Nephrology, Dialysis and Transplantation Unit, IRCCS Istituto Giannina Gaslini, Genoa, Italy; 11 Department of Clinical Immunology and Internal Medicine, CHU of Caen Normandie, Caen, France; 12 Normandie Universite, UNICAEN, CHU de Caen Normandie, Caen, France; 13 Department of Radiology, Pitié-Salpȇtrière Hospital, Sorbonne University, Assistance Publique-Hôpitaux de Paris, Paris, France; 14 Internal Medicine Department 2, Institut E3M, French Reference Centre for Histiocytosis, CIMI INSERM-UMRS 1135, Pitié-Salpȇtrière Hospital, Sorbonne University, Assistance Publique-Hôpitaux de Paris, Paris, France

**Keywords:** BRAF inhibitors, Erdheim-Chester disease, histiocytosis, kidney transplantation, mTOR inhibitors, pediatric histiocytosis

## Introduction

**Figure 1:**
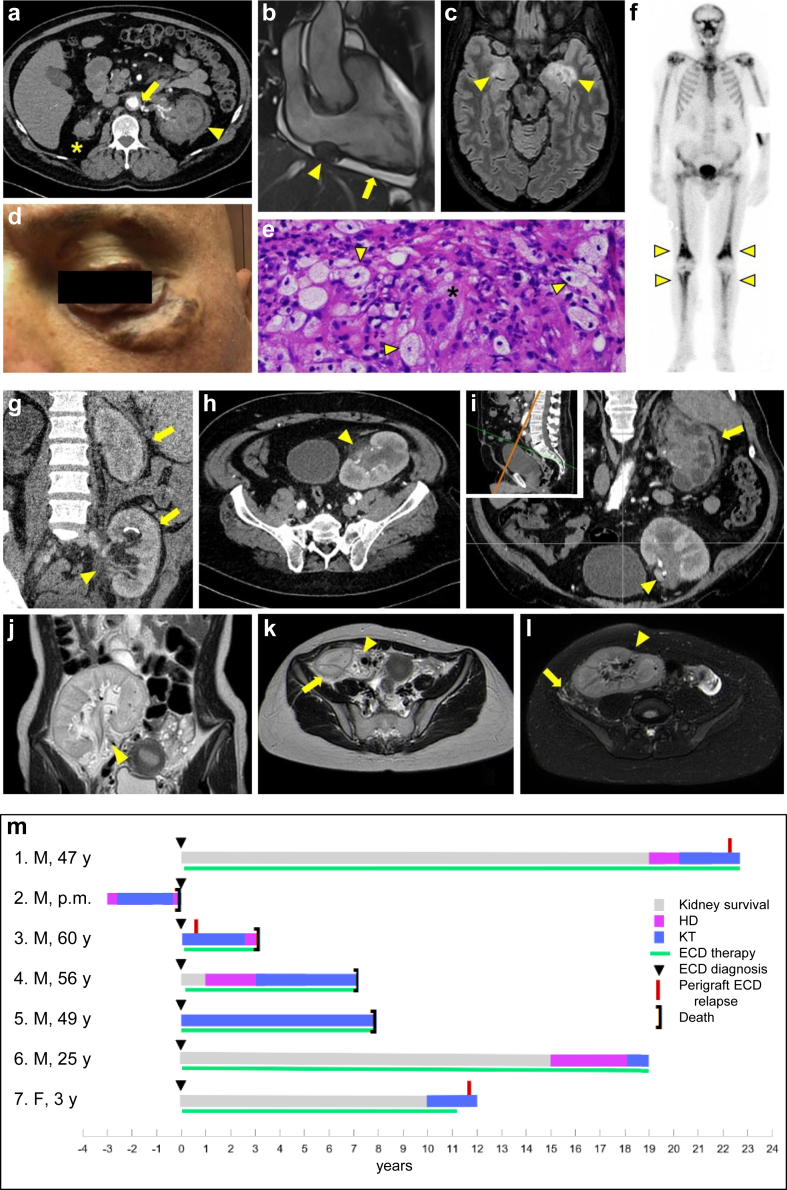
Imaging and histological features of Erdheim-Chester disease. (a–f) Show typical systemic ECD involvement; (a) Abdominal computed tomography of patient #1. This axial view scan (arterial phase) shows infiltration of the perirenal space of the left native kidney, which shows the typical “hairy kidney” appearance (arrowhead); the right native kidney is atrophic (asterisk) and periaortic infiltration can also be seen (arrow). (b) Nodular lesion in the right atrioventricular sulcus inseparable from the pericardium (arrowhead) and pericardial effusion (arrow) on cardiac magnetic resonance imaging of patient #6. (c) Hyperintense lesions in the temporal cerebral lobes on T2-weighted-Fluid-Attenuated Inversion Recovery image in brain magnetic resonance imaging of patient #3. (d) Eyelid xanthelasma of patient #1. (e) Bone biopsy of patient #3 showing foamy histiocytes (arrowheads) and a Touton multinucleated giant cell (asterisk) surrounded by fibrosis and inflammatory cells. (f) Increased and symmetrical ^99^Tc uptake in the metaphysis of long bones (distal femur and proximal tibia) on bone scintigraphy of patient #3. (g–l) Illustrate images of perigraft recurrence of ECD. (g) Abdominal computed tomography of patient #3; a coronal scan in early venous phase shows the kidney graft in the left iliac fossa with perigraft (lower arrow) and renal sinus (arrowhead) infiltration; there is also perirenal infiltration of the left native kidney (upper arrow), indicating recurrence of ECD. (h and i) Abdominal computed tomography of patient #1. The (h) axial and (i) oblique scans, obtained in an early venous phase, show the graft in the left iliac fossa with infiltration of the renal sinus (arrowheads) indicating probable recurrence of ECD, along with infiltration of perirenal space of the left native kidney (arrow). (i) In the inset, the orange line denotes the orientation of the oblique scan. Other images of the same patient are published in Chazal *et al.*^2^*Kidney Int* 2023. (j–l) Abdominal magnetic resonance imaging of patient #7. The coronal and axial T2-weighted turbo spin echo (T2w-TSE) scans (j and k) show the graft in the right iliac fossa with signs of infiltration of graft sinus (arrowheads) and perirenal space (arrows), better evident on T2-weighted image with fat saturation (T2-SPAIR sequence) (l). (m) Swimmer plot showing the course of ECD and kidney outcome of the 7 patients included in the study. ECD, Erdheim-Chester disease; F, female; HD, hemodialysis; KT, kidney transplantation; M, male; p.m., postmortem.

Erdheim-Chester disease (ECD) is a rare histiocytosis driven by somatic mutations involving genes of the MAPK pathway (e.g., *BRAF*^*V600E*^).^1^ Bilateral perirenal and retroperitoneal involvement occurs in approximately 70% of cases and typically consists of infiltration of the perirenal space (“hairy kidney” appearance), the pelvis, the proximal ureter, and the renal vascular peduncle^2^ (Figure 1a–f). It is often asymptomatic but may cause abdominal or lumbar pain and renovascular hypertension. Unrecognized long-standing perirenal infiltration can lead to obstructive or ischemic nephropathy, ultimately causing chronic kidney disease and, in some cases, kidney failure.^2^, ^3^, ^4^, ^5^, ^6^, ^7^ Despite its clonal pathogenesis, ECD does not usually worsen during immunosuppression (which has actually been used to treat it in previous years), so kidney transplantation (KT) remains a feasible renal replacement therapy option. There is very limited data on graft and patient outcomes after KT, because only 1 case has been reported so far.^7^ Herein, we analyzed the clinical presentation and outcome of KT in a series of 7 patients with ECD.

## Results

### Patients’ Characteristics

Seven patients (1 female child and 6 male adults) with ECD who received KT for ECD-related kidney failure were identified (Table 1). One of them was previously described in a case report (patient #4),^7^ and 2 (patients #3, #7) were included in aggregated cohorts.^2^^,^^8^

**Table 1:** Demographic and clinical data, characteristics and timing of RRT, therapy and outcome of patients included in the study

Patient #	Age at ECD symptoms onset (yr)	Comorbidity	Pattern of kidney involvement	Extrarenal manifestations	Somatic mutations	Age at kidney failure (yr), type of RRT	Age at KT (yr), type of donor	Pre-KT ECD therapy	KT therapy	Post-KT follow-up
Sex	Age at ECD diagnosis (yr)								Post-KT ECD therapy	Outcome at last follow-up
#1	NA	Hypertension	Obstructive nephropathy, renal vascular disease	Periaortic, long bone, skin	*BRAF*^V600E^	66, HD	67, living donor	IFN a, anakinra, canakinumab, GC	Induction: NA Maintenance: TAC, MMF, GC	70 yr: perigraft ECD recurrence.
M	47								Anakinra	70 yr: alive eGFR 56 ml/min per 1.73 m^2^
#2	63	Hypertension	Bilateral obstructive nephropathy	CNS, thyroid, heart, lung, lymph node, adrenal, testis, long bone	NA	64, HD	64, living donor	none	Pre-KT: plasmapheresis, i.v. Ig Induction: antithymocyte globulins Maintenance: TAC, MMF, GC	General clinical deterioration 67 yr: kidney failure, HD
M	Post-mortem								none	67 yr: death
#3	60	Heart failure Hypertension Hypercholesterolemia T2D Myelofibrosis	Renal vascular disease	CNS, heart, lung, periaortic, long bone	*BRAF*^V600E^	60, KT	60, DBD (combined hearth-KT)	none	Induction: NA Maintenance: TAC, MMF, GC	61 yr: peri-graft ECD recurrence 61 yrr: sepsis 62 y: kidney failure, HD
M	60								Cobimetinib	63 yr: death
#4	56	None	Obstructive nephropathy, renal vascular disease	Long bone	*BRAF*^V600E^	57, HD	59, DBD	Vemurafenib	Induction: basiliximab Maintenance: TAC, MMF, AZA, GC	59 yr: acute T cell-mediated rejection (Banff IIA) 63 yr: sepsis
M	56								Vemurafenib	63 yr: death
#5	47	NA	Bilateral obstructive nephropathy	Orbit, CNS, long bone	*BRAF*^V600E^	49, KT	49, living donor	Vemurafenib	Induction: NA Maintenance: rapamycin, MMF, GC	Recurrent urinary tract infections 57 yr: sepsis
M	49								Vemurafenib	57 yr: death
#6	24	Obesity	Obstructive nephropathy, renal vascular disease	Orbit, heart, periaortic, long bone	*BRAF*^V600E^	40, HD	43, DBD	Vemurafenib, IFN a, MTX, infliximab, tocilizumab, GC	Induction: basiliximab Maintenance: TAC, everolimus, GC	DGF
M	25								Everolimus, tocilizumab	44 yr: alive eGFR 24 ml/min per 1.73 m^2^
#7	1	None	Bilateral obstructive nephropathy	Orbit, lymph node, long bone, skin	None	13, KT	13, DBD	Everolimus, IFN a	Induction: basiliximab Maintenance: TAC, MMF, GC	15 yr: perigraft ECD recurrence
F	3								Everolimus	15 yr: alive, eGFR 52 ml/min per 1.73 m^2^

AZA, azathioprine; CNS, central nervous system; CyA, cyclosporine; DBD, deceased brain-dead donor; DGF, delayed graft function; ECD, Erdheim-Chester disease; eGFR, estimated glomerular filtration rate (according to the Chronic Kidney Disease-Epidemiology Collaboration equation for adults and CKiD-U25 for children); F, female; GC, glucocorticoids; HD, hemodialysis; IFN a: interferon-alpha; KT, kidney transplantation; M, male; MMF, mycophenolate mofetil; MTX, methotrexate; NA, not available; T2D, type 2 diabetes; TAC, tacrolimus; RRT, renal replacement therapy.

### ECD Diagnosis and Pretransplant Manifestations

The median age at ECD diagnosis was 49 (range: 4–67) years and diagnosis occurred a median time of 1.5 years after the onset of the first symptoms attributable to the disease (range 0–4). In patient #2 the diagnosis was made postmortem; whereas in patient #5 it was by kidney histological examination following bilateral nephrectomy, which was performed in conjunction with a KT. Patient #3 underwent simultaneous heart transplantation and KT, and was diagnosed after analyzing the native explanted heart. Patient #4 was diagnosed with ECD after developing kidney failure, whereas the remaining 3 (#1, #6, and #7) had ECD with mild-to-moderate chronic kidney disease that slowly progressed to kidney failure (median time from ECD diagnosis to kidney failure: 15 years, range: 10–19 years). Five of the 6 tested patients harbored the *BRAF*^V600E^ mutation on ECD tissue biopsies (Table 1).

Kidney involvement included obstructive nephropathy (*n* = 6, 86%) and/or renal vascular disease because of renal artery stenosis (*n* = 4, 57%) and 3 cases showed the typical “hairy kidney” appearance. Extrarenal manifestations included long-bone (*n* = 7, 100%), cardiac (*n* = 3, 43%), neurological (*n* = 3, 43%), periaortic (*n* = 3, 43%), retro-orbital (*n* = 3, 43%), cutaneous (*n* = 2, 29%) and lymph node (*n* = 2, 29%) involvement (Table 1).

The diagnosis period ranged from 2001 to 2018, so ECD therapy reflected the evolving knowledge and included different agents, particularly BRAF inhibitors (*n* = 3, 43%), MEK inhibitors (*n* = 1, 14%), interferon-alpha (*n* = 3, 43%), mTOR inhibitors (*n* = 2, 29%), and anticytokine drugs (*n* = 2, 29%) (Figure 1m; Table 1). Ureteral or vascular stents were placed in 2 patients but failed to improve renal function or hypertension in both cases.

### Posttransplant Management

Three patients (#3, #5, and #7) received preemptive KT, whereas 4 underwent hemodialysis (median duration: 1.5 years) before KT. Living donors were available for 3 patients (patients #1, #2, and #5), whereas the remaining 4 were transplanted from brain-dead donors. Patient #2 had a non–human leukocyte antigen positive crossmatch with his donor and was therefore treated with plasmapheresis and i.v. Ig.

Following local protocols, 5 patients (72%) received standard posttransplant immunosuppression with glucocorticoids, mycophenolate, and calcineurin inhibitors; a patient switched from mycophenolate to azathioprine after a few months because of cost; and 2 patients (29%) received glucocorticoids, calcineurin inhibitors, and mTOR inhibitors. Induction was obtained with antithymocyte globulins (*n* = 1, 25%) or basiliximab (*n* = 3, 75%) (Table 1); data on induction was not available for the remaining 3 patients.

Only patients #1, #4, #6, and #7 underwent KT during a period of disease remission and following prolonged and appropriate therapy for ECD; this was maintained after KT in all but patient #7, who discontinued it after 9 years of remission (1 year after KT). In the remaining cases, ECD therapy was started after KT or shortly before it, except for patient #2 who never received it (Figure 1m). Patient #6 switched from a BRAF inhibitor to an mTOR inhibitor plus tocilizumab.

### Outcome

After transplantation, patient #6 showed delayed graft function, whereas patient #4 developed acute cell-mediated rejection (Banff IIA) 2 months after KT. Three patients had imaging findings suggestive of ECD relapse involving the perigraft area (especially the renal sinus) (Figure 1g–l) 6 months (patient #3) and 3 years (patients #1 and #7) after KT; this was detected using computed tomography or magnetic resonance imaging (Figure 1) but in none of these cases perirenal biopsy was performed. Moreover, perigraft infiltration by foamy histocytes was detected in patient #2’s autopsy. Patient #3, who had undergone simultaneous heart transplantation and KT, had a relapse that involved both grafts.

After a median of 3.5 years posttransplant (range: 1-8 years), 4 patients died (patients #3, #4, and #5 for sepsis, patient #2 for ECD progression), 2 with a functioning graft (Figure 1m). The remaining 3 had functioning grafts at last follow-up but experienced chronic graft dysfunction (stages III and IV according to NKF-KDOQI), apparently unrelated to ECD (Supplementary Figure S1).

## Discussion

Most patients with ECD present with perirenal involvement, which typically leads to chronic kidney disease. However, given the slowly progressive course of chronic kidney disease and the high mortality associated with ECD, only a minority of patients eventually progress to kidney failure. KT can be considered an appropriate renal replacement therapy modality in selected patients with ECD, and the clonal nature of the disease should not, by itself, preclude timely referral for transplantation. However, access to KT in patients with ECD may be limited by cardiac involvement or by surgical or vascular challenges, particularly in the presence of periiliac infiltration. Therefore, our unique cohort provides valuable insights into the feasibility and outcomes of KT in this disease.

The management of ECD after transplant is further complicated by the delicate balance of immunosuppression. By the time patients with ECD reach KT, most already have a long exposure to therapies, which may include immunosuppressants; these, when combined with antirejection regimens, can result in an excessive risk of infections. Sepsis was, in fact, the leading cause of mortality in our cohort. Alternatively, discontinuing therapy for ECD in an attempt to reduce the overall immunosuppressive burden may increase the risk of disease recurrence, even many years after the initial diagnosis. This balance probably plays a decisive role in graft and patient outcomes, in addition to the challenges of late diagnosis and only partially effective therapies. Another challenge for clinicians in this setting is choosing the appropriate drug and determining the right timing for its introduction or discontinuation. For instance, alpha-interferon, used to control ECD, is contraindicated in transplant recipients because of its potential to induce graft rejection; thus, it should be replaced in advance with a more suitable agent when a KT is planned. mTOR inhibitors, effective both in managing ECD and as antirejection therapy, may theoretically represent a good option, offering patients both antiproliferative and immunosuppressive benefits.^9^

Perigraft disease relapses are possible (and seemingly frequent) even in the absence of systemic disease reactivation. The short follow-up in our patients prevents determining if perigraft relapse affects kidney function. However, the preferential sites of infiltration mirror those typically observed in the native kidney (renal capsule and peri-sinusal region), and it is therefore plausible that the clinical consequences may be comparable. Of note, the observation that ECD may relapse around the kidney graft, which is located in the extraperitoneal space—an area usually spared by ECD—raises intriguing questions on ECD pathogenesis and tissue tropism of the histiocytic infiltration. This finding suggests that local microenvironmental factors introduced during graft implantation, such as perisinusal fat, might create a permissive niche for the recruitment of mutated circulating monocytes/macrophages. Although purely speculative, this hypothesis highlights the need for a deeper understanding of the determinants of histiocyte homing and the biological characteristics that make specific tissues more vulnerable to ECD involvement. To improve patient and graft outcomes, achieving ECD remission before undergoing KT, as well as continuing ECD-specific therapy after transplant seem reasonable strategies.

In conclusion, our study provides the first description of KT in a series of patients with ECD, suggesting that KT is feasible in selected cases but associated with considerable risks, which may be mitigated by optimal management of the underlying disease and prompt intervention in the event of posttransplant complications.

## Disclosure

All the authors declared no competing interests.

## Supplementary Material

Supplementary File (PDF) Supplementary Methods. Supplementary References. Figure S1. Estimated glomerular filtration rate according to CKD-EPI (or CKiD U25 formula in patient 7) at 3, 6, 12. and 24 months since kidney transplantation.

## Footnotes

Supplementary File (PDF)

**Supplementary Methods.**

**Supplementary References.**
**Figure S1.** Estimated glomerular filtration rate according to CKD-EPI (or CKiD U25 formula in patient 7) at 3, 6, 12. and 24 months since kidney transplantation. Data of patient #5 are missing.

## References

[1] Pegoraro F., Papo M., Maniscalco V., Charlotte F., Haroche J., Vaglio A. (2020). Erdheim-Chester disease: a rapidly evolving disease model. Leukemia.

[2] Chazal T., Pegoraro F., Manari G. (2023). Clinical phenotypes and long-term outcome of kidney involvement in Erdheim-Chester histiocytosis. Kidney Int.

[3] Sanchez J.E., Mora C., Macia M., Navarro J.F. (2010). Erdheim-Chester disease as cause of end-stage renal failure: a case report and review of the literature. Int Urol Nephrol.

[4] Konishi R., Morinishi T., Takaori K., Iwamoto Y., Kondo M., Maeda S. (2022). A long-term survival case of Erdheim-Chester disease on maintenance hemodialysis. CEN Case Rep.

[5] Tsukamoto M., Akahane M., Nangaku M. (2012). Image of Erdheim-Chester disease requiring hemodialysis. Clin Exp Nephrol.

[6] Chen M.T., Wang S.M., Lin S.Y. (2012). Pericardial effusion as a crucial presentation of Erdheim-Chester disease in a hemodialysis patient: an overlooked diagnosis. Clin Nephrol.

[7] Yoo J., Gunsteen C., Patel S. (2020). Kidney transplantation for Erdheim-Chester disease. Case Rep Transplant.

[8] Pegoraro F., Mazzariol M., Trambusti I. (2023). Childhood-onset Erdheim-Chester disease in the molecular era: clinical phenotypes and long-term outcomes of 21 patients. Blood.

[9] Pegoraro F., Maniscalco V., Peyronel F. (2020). Long-term follow-up of mTOR inhibition for Erdheim-Chester disease. Blood.

