# Supplementary material for: Kidney Transplantation in Patients With Erdheim-Chester Disease
Source: Kidney Int Rep. 2026 Jan 2;11(3):103763. doi: 10.1016/j.ekir.2025.103763 (PMC12874406; doi:10.1016/j.ekir.2025.103763)
Supplement: Supplementary File (PDF) — Supplementary Methods. Supplementary References. Figure S1. Estimated glomerular filtration rate according to CKD-EPI (or CKiD U25 formula in patient 7) at 3, 6, 12. and 24 months since kidney transplantation. [file mmc1.pdf]

Supplementary Materials

Supplementary Methods

We retrospectively collected data on KT in patients with ECD across the ECD Global Alliance Care Centers and patient representative network (www.erdheim-chester.org). We also reviewed the literature and found just one case report,**[7]** thus we contacted its corresponding author for updated follow-up data.

We included patients diagnosed with ECD following the latest consensus guidelines.**[S1]** Data on disease presentation, phenotypes of kidney involvement, treatments, and long-term graft and patient outcome were collected. The study was conducted in accordance with the Declaration of Helsinki and its amendments**.[S2]**

Supplementary Figure S1. eGFR according to CKD-EPI (or CKiD U25 formula in patient 7) at three, six, 12 and 24 months since KT. Data of patient #5 are missing.

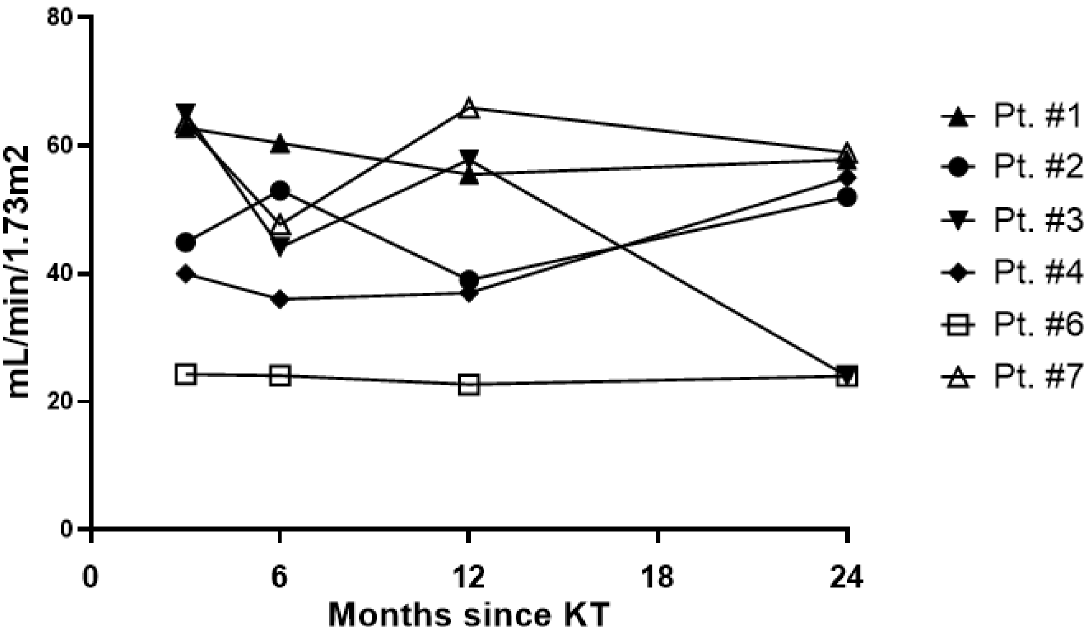

Abbreviations: KT: kidney transplantation; Pt: patient

Supplementary References

[S1] Goyal G, Heaney ML, Collin M, et al. Erdheim-Chester disease: consensus recommendations for evaluation, diagnosis, and treatment in the molecular era. *Blood*. 2020;135(22):1929–45.

[S2] World Medical Association. World Medical Association Declaration of Helsinki: ethical principles for medical research involving human subjects. *JAMA*. 2013;310(20):2191-4.
